# Isolation of a novel mutant gene for soil-surface rooting in rice (*Oryza sativa* L.)

**DOI:** 10.1186/1939-8433-6-30

**Published:** 2013-11-20

**Authors:** Eiko Hanzawa, Kazuhiro Sasaki, Shinsei Nagai, Mitsuhiro Obara, Yoshimichi Fukuta, Yusaku Uga, Akio Miyao, Hirohiko Hirochika, Atsushi Higashitani, Masahiko Maekawa, Tadashi Sato

**Affiliations:** 1 Graduate School of Life Sciences, Tohoku University, 2-1-1 Katahira, Aoba-ku, Sendai, Miyagi 980-8577, Japan; 2 Present address: Plant Breeding, Genetics and Biotechnology Division, International Rice Research Institute, DAPO Box 7777, Metro Manila, Philippines; 3 Japan International Research Center for Agricultural Sciences, 1-1 Ohwashi, Tsukuba, Ibaraki 305-8686, Japan; 4 National Institute of Agrobiological Sciences, 2-1-2 Kannondai, Tsukuba, Ibaraki 305-8602, Japan; 5 Institute of Plant Science and Resources, Okayama University, 2-20-1, Kurashiki, Okayama 710-0046, Japan; 6 RIKEN Innovation Center, Ion Beam Breeding Laboratory, 2-1 Hirosawa, Wako, Saitama 351-0198, Japan

**Keywords:** Root system architecture, Gene isolation, Soil-surface rooting, Shallow rooting, Gravitropic response

## Abstract

**Background:**

Root system architecture is an important trait affecting the uptake of nutrients and water by crops. Shallower root systems preferentially take up nutrients from the topsoil and help avoid unfavorable environments in deeper soil layers. We have found a soil-surface rooting mutant from an M_2_ population that was regenerated from seed calli of a *japonica* rice cultivar, Nipponbare. In this study, we examined the genetic and physiological characteristics of this mutant.

**Results:**

The primary roots of the mutant showed no gravitropic response from the seedling stage on, whereas the gravitropic response of the shoots was normal. Segregation analyses by using an F_2_ population derived from a cross between the soil-surface rooting mutant and wild-type Nipponbare indicated that the trait was controlled by a single recessive gene, designated as *sor1*. Fine mapping by using an F_2_ population derived from a cross between the mutant and an *indica* rice cultivar, Kasalath, revealed that *sor1* was located within a 136-kb region between the simple sequence repeat markers *RM16254* and *2935-6* on the terminal region of the short arm of chromosome 4, where 13 putative open reading frames (ORFs) were found. We sequenced these ORFs and detected a 33-bp deletion in one of them, *Os04g0101800*. Transgenic plants of the mutant transformed with the genomic fragment carrying the *Os04g0101800* sequence from Nipponbare showed normal gravitropic responses and no soil-surface rooting.

**Conclusion:**

These results suggest that *sor1*, a rice mutant causing soil-surface rooting and altered root gravitropic response, is allelic to *Os04g0101800*, and that a 33-bp deletion in the coding region of this gene causes the mutant phenotypes.

## Background

Root system architecture is an important trait for plant growth and crop yield because it affects the uptake of nutrients and water, which are unevenly distributed in the soil. Diversity of the vertical distribution of the root systems of different species allows efficient maximization of plant growth and crop yields in unfavorable environments with poor resource mobility, such as infertile or arid fields (Hodge et al. 2009; Rich and Watt 2013). Shallow root systems enhance phosphorus acquisition in common bean (Ge et al. 2000; Liao et al. 2004; Lynch 2011) and wheat (Manske et al. 2000). Phosphorus-efficient bean cultivars have shallower basal roots and more adventitious rooting in the topsoil than other bean cultivars (Lynch and Brown 2001). In teosintes, adventitious root formation at the soil surface may provide an alternative growth strategy to cope with soil flooding or waterlogging (Mano et al. 2005). Therefore, shallower root systems preferentially take up nutrients from the topsoil and help to avoid unfavorable environments in deeper soil layers.

When submerged, Indonesian rice (*Oryza sativa* L.) cultivars that belong to the Bulu ecotype develop thick primary roots above and near the soil surface (Lafitte et al. 2001; Ueno and Sato 1989, 1992). These authors suggested that soil-surface and shallow roots contribute to the avoidance of hypoxic soils in rice. Rice superficial roots develop near the soil surface from the beginning of the spikelet differentiation stage to the fully ripe stage (Morita and Yamazaki 1993). The fresh weight of superficial roots is positively correlated with yields in paddy fields (Morita and Yamazaki 1993). Thus, soil-surface and shallow roots may contribute to the avoidance of hypoxic environments and promote rice growth.

Genetic analyses of root system architecture have been performed in a variety of rice lines; however, no genes controlling soil-surface rooting have been isolated by using natural variation. A study of 81 diverse genotypes in rice revealed two DNA molecular markers associated with shallow and deep rooting on chromosomes 9 and 10, respectively (Chaitra et al. 2006). Uga et al. (2012) identified four quantitative trait loci (QTLs) for soil-surface rooting on chromosomes 3, 4, 6, and 7 by using recombinant inbred lines derived from a cross between an Indonesian rice cultivar with soil-surface roots and a Japanese cultivar without such roots. The use of mutants is an alternative approach to isolate causal genes for traits of interest. Rice mutants have been isolated for the following root morphological characteristics: inhibition of root growth, absence of crown roots, short lateral roots or absence of lateral roots, short root hairs, and hairless roots (Chhun et al. 2003; Debi et al. 2003, 2005; Hao and Ichii 1999; Ichii and Ishikawa 1997; Inukai et al. 2001; Iwao et al. 2005; Kim et al. 2007; Suzuki et al. 2003; Yuo et al. 2009, 2011). Although many rice mutants for the above traits have been reported, no causal genes for soil-surface rooting have been cloned. Here, we describe a rice mutant with thick primary roots that develop above and immediately under the soil surface. The purpose of this study was to isolate and characterize the mutant gene responsible for soil-surface rooting.

## Results

### Evaluation of the mutant phenotype

**Figure 1:**
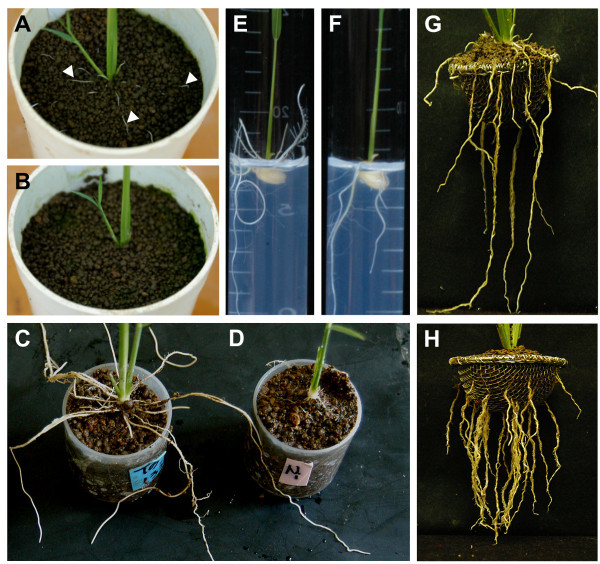
**Differences in rooting between the *sor1 *mutant (A, C, E, and G) and wild-type Nipponbare (B, D, F, and H).** Plants were grown in pots under waterlogged conditions **(A and B)**, by the cup method **(C and D)**, by the glass tube method **(E and F)**, or by the basket method **(G and H)**. White arrowheads in **(A)** indicate soil-surface roots.

To evaluate the soil-surface rooting phenotype of the mutant, we used four different methods: the pot method, the cup method, the glass tube method, and the basket method. Under submerged conditions, the roots of all mutant plants elongated on or just below the soil surface in plant pots (Figure 1A); however, no wild-type Nipponbare plants (WT) had such roots during the seedling stage (Figure 1B). The cup method showed that mutant plants had many soil-surface roots (Figure 1C); however, WT had very few such roots (Figure 1D). The glass tube method showed that mutant plants had many primary roots elongated on the upper side of the agar surface (Figure 1E); however, WT had no such roots (Figure 1F). The basket method showed that mutant plants had many soil-surface roots (Figure 1G); however, WT had few soil-surface roots (Figure 1H). These results revealed that our mutant is a novel mutant for soil-surface rooting.

### Distribution of primary roots assessed by basket method

**Figure 2:**
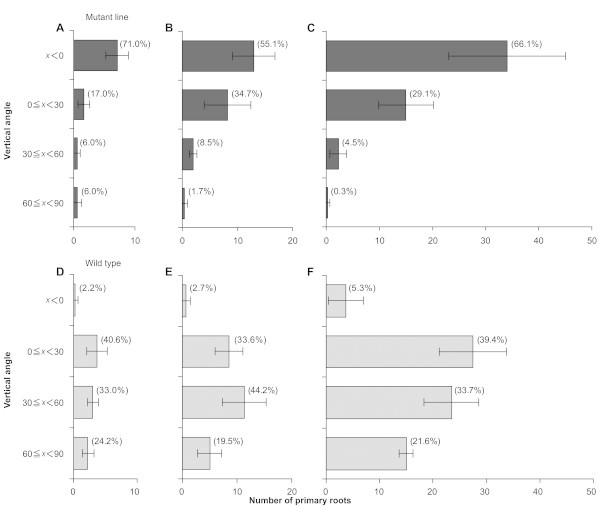
**Distribution of primary roots between vertical-angle zones in the *sor1 *mutant (A, B, and C) and wild-type Nipponbare (D, E, and F), grown under waterlogged conditions by using the basket method.** The percentages of primary roots in each angle category were assessed at the third **(A and D)**, fifth **(B and E)**, and seventh **(C and F)** leaf stages (*n* = 9), and are shown in parentheses. Error bars indicate SE.

The primary roots of the mutant plants tended to be distributed horizontally at the third (Figure 2A and D), fifth (Figure 2B and E), and seventh leaf stages (Figure 2C and F). More than 88% of the primary roots in the mutant (Figure 2A–C), but less than 45% in the WT (Figure 2D–F) had a vertical growth angle of < 30° (soil surface = vertical angle of 0°) at any of the examined leaf stages. These results suggest that the primary roots of the mutants elongated on or near the soil surface from early seedling stages on, whereas those of WT tended to elongate toward the direction of gravity.

### Gravitropic responses of roots and shoots

**Figure 3:**
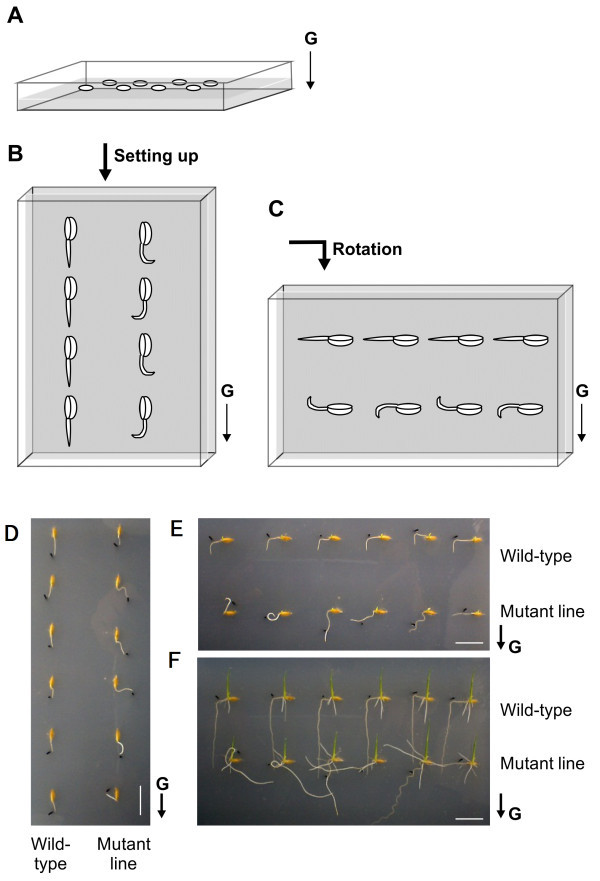
**Gravitropic response assay for shoots and primary roots at the seedling stage.** A diagram illustrating the assay is shown in **A–C**. Germinated seeds are sown just under the surface of 1.5% agar in plastic trays with the embryo placed upward **(A)**. Trays were set on their short sides 18 h after sowing **(B)** and rotated 90° clockwise 10 h later **(C)**. Differences in root gravitropic responses in mutant and Nipponbare seedlings 10 h after setting the tray on a short edge **(D)**, 10 h after rotation **(E)**, and 76 h after rotation **(F)**. Black dots in **D–F** mark the location of seminal root tips just before the rotation. Scale bars, 2 cm. The direction of gravity **(G)** is indicated by arrows.

The gravitropic responses of seminal roots in WT and mutant seeds were evaluated in an agar tray assay (Figure 3A–C). Prior to rotation, the mutant roots were growing in random directions, whereas WT roots elongated along the direction of gravity (Figure 3D). After the trays were rotated 90°, the mutant roots continued to extend randomly, whereas those of WT bent in the direction of gravity within 10 h (Figure 3E). Although the direction of the seminal roots of the mutants was irregular before and after rotation, their shoots grew normally against the direction of gravity (Figure 3F). Furthermore, the mutant crown roots extended randomly, whereas the WT crown roots grew in the direction of gravity. These results suggest that the primary roots of the mutant lack a gravitropic response from the seedling stage on, whereas the shoots show normal gravitropic response.

### Relationship between soil-surface rooting and gravitropic response

To determine whether the soil-surface rooting and the lack of root gravitropic response observed in the mutant are controlled by the same gene, we performed segregation analyses by using 85 F_2_ plants derived from a cross between the mutant and WT Nipponbare (Table 1). The gravitropic responses of seminal roots of each F_2_ plant were evaluated in the agar tray assay (Figure 3A–C). The primary roots of 60 F_2_ plants responded to gravity similarly to WT Nipponbare roots. In contrast, the primary roots of the remaining 25 plants showed gravity-insensitivity that was similar to that of the mutant. The ratio of gravity-sensitive plants to insensitive plants was not significantly different from 3:1 (χ^*2*^ = 0.797, *P* = 0.372).

**Table 1:** **Segregation of gravitropic responses of primary roots and the ratio of soil-surface roots to total roots in the F**_**2 **_**population**

**Plants**	**Number of plants**	Soil-surface root ratio (%)*		
		**Mean ± S.D.**	**Minimum**	**Maximum**
Mutant line	9	64.4 ± 17.3	26.5	82.7
Gravity-insensitive F_2_ plants	25	69.1 ± 10.1	52.6	85.0
Gravity-sensitive F_2_ plants	60	2.8 ± 3.0	0.0	11.4
Nipponbare	9	5.1 ± 4.4	0.0	14.1

*The soil-surface rooting ratio was calculated as (the number of primary roots emerged at *x* < 0° / the total number of primary roots) × 100.

In the 60 F_2_ plants that had gravity-sensitive roots, the soil-surface rooting ratio was 11.4% or less (Table 1). In contrast, the remaining 25 plants had a soil-surface rooting ratio of 52.6% or more. These results suggest that soil-surface rooting at the seedling stage is controlled by a single recessive gene that also controls the lack of response to gravity beginning at germination. We designated the recessive mutant gene as *sor1* (*soil-surface rooting 1*) and the corresponding locus as *SOR1*.

### Mapping of the mutant sor1 gene

**Figure 4:**
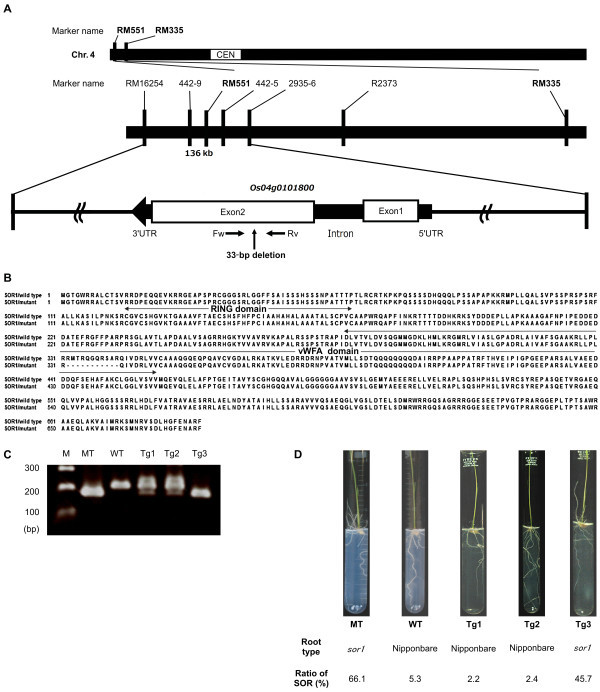
**Map position and complementation of the candidate gene for soil-surface rooting. (A)** Fine mapping of the *SOR1* locus, carried out by using SOR-type plants, indicated that the DNA marker *RM551* on chromosome 4 co-segregated with soil-surface rooting. The *SOR1* locus was identified in a 136-kb region between markers *RM16254* and *2935*–*6*. The *Os04g0101800* gene is shown as a black arrow, with white boxes indicating the exons. The second exon of the *sor1* mutant allele contains a 33-bp genomic deletion. FW and RV, specific primers used to confirm the deletion. **(B)** Alignment of SOR1 (wild-type and mutant) amino acid sequences. The positions of the zinc finger (RING finger) domain (aa124–170) and the vWFA (von Willebrand factor type **A**) domain (aa279–460) are indicated above the sequences. The 11-aa deletion (aa332–342) in the vWFA domain of the mutant is shown with dashes (-). **(C)** Confirmation of the genomic deletion in the mutant by analysis of the PCR amplicon size in wild-type (WT), mutant (MT), and transgenic plants (Tg1–3). Tg1 and Tg2 were transformed with a6677-bp genomic fragment containing *Os04g0101800*; Tg3 was transformed with an empty vector. M, DNA marker. **(D)** Phenotyping of soil-surface rooting by the glass tube method (top line) and ratio of soil-surface rooting (SOR) by the basket method (bottom line) in the mutant, wild-type, and transgenic plants.

We used the cup method to determine the soil-surface rooting ratio of F_2_ plants derived from a cross between the mutant and Kasalath, which has a soil-surface rooting ratio similar to that of Nipponbare (data not shown). Among 103 F_2_ plants, 19 had soil-surface rooting ratios similar to those of the mutant, and the remaining 84 plants were normal. The ratio of Nipponbare-type (normal) to SOR-type (soil-surface rooting) plants was not significantly different from 3:1 (χ^*2*^ = 2.359, *P* = 0.125). Rough mapping was carried out by using the 19 SOR-type plants and 70 DNA markers known to be polymorphic between Nipponbare and Kasalath (Additional file 1). *RM551* in the terminal region of the short arm of chromosome 4 (Figure 4A) was the only marker for which the Nipponbare allele was detected in all SOR-type plants. This result indicated that the Nipponbare allele of *RM551* co-segregated with the *sor1* mutant allele.

To narrow the candidate region for *sor1*, 647 F_2_ plants were used for fine mapping. The phenotype of each F_2_ plant was evaluated individually by the glass tube method (which is less labor-intensive than the cup method). As in previous experiments (Figure 1E), the mutant roots elongated on the agar surface and extended against the direction of gravity, whereas the roots of Kasalath elongated into the agar along the direction of gravity (data not shown). A total of 498 and 149 plants were of the Nipponbare-type and SOR-type, respectively, and the segregation ratio was not significantly different from 3:1 (χ^*2*^ = 1.340, *P* = 0.247). Fine-mapping analyses with seven markers on chromosome 4 (*RM16254*, *442–9*, *RM551*, *442–5*, *2935–6*, *R2373*, and *RM335*; Additional file 2) were performed to identify the markers flanking the *sor1* gene. Six recombination events were detected between *RM16254* and *442*–*9*, and one recombination event was detected between *442*–*5* and *2935*–*6* (Figure 4A). The Nipponbare alleles of markers *442*–*9*, *RM551*, and *442*–*5* co-segregated with the *sor1* mutant. These results indicated that *sor1* is located between markers *RM16254* and *2935*–*6*. The interval defined by *RM16254* and *2935*–*6* spans approximately 136 kb, according to the Nipponbare genome sequence (IRGSP build 5; International Rice Genome Sequencing Project 2008 (http://rgp.dna.affrc.go.jp/E/IRGSP/Build5/build5.html).

### Sequence analysis of SOR1

According to the Rice Annotation Project Database (http://rapdb.dna.affrc.go.jp/), a total of 13 putative ORFs are located in the 136-kb *sor1* candidate genomic region. To determine whether any of these genes are mutated relative to wild-type Nipponbare, we sequenced the genomic DNAs of all 13 candidate genes from Nipponbare and the mutant. A 33-bp deletion was detected in the *Os04g0101800* ORF, whereas no polymorphisms were detected in the other 12 genes. The protein encoded by *Os04g0101800*, which we named SOR1, has two domains: a zinc finger (RING) domain (aa124–170) and a von Willebrand factor type A (vWFA) domain (aa279–460) (Figure 4B). The deletion in *sor1* resulted in a loss of 11 amino acids (aa332–342) in the vWFA domain (Figure 4B). On the basis of these data, we concluded that *Os04g0101800* was the most likely candidate for the gene mutated in *sor1*.

### Complementation of the candidate gene for sor1

To verify that the deletion in *Os04g0101800* was responsible for the *sor1* mutant phenotype, we carried out a complementation test. We generated transgenic lines by transformation of the mutant with a 6677-bp genomic DNA fragment from WT Nipponbare containing the entire *Os04g0101800* coding region. The presence of the *Os04g0101800* transgene was confirmed in two independent transgenic lines, Tg1 and Tg2, by detecting PCR fragments of the same length as in WT (Figure 4C). These lines showed normal seminal root direction when assessed by the glass tube method, and their soil-surface rooting (assessed by the basket method) was similar to that of Nipponbare (Figure 4D). In contrast, transgenic lines created by using an empty vector showed the *sor1* phenotype for both seminal root direction and soil-surface rooting (Tg3 in Figure 4C and D). Thus, the complementation analysis demonstrated that the 33-bp deletion in *Os04g0101800* is responsible for the mutant phenotypes.

### Phylogenetic analysis of SOR1

**Figure 5:**
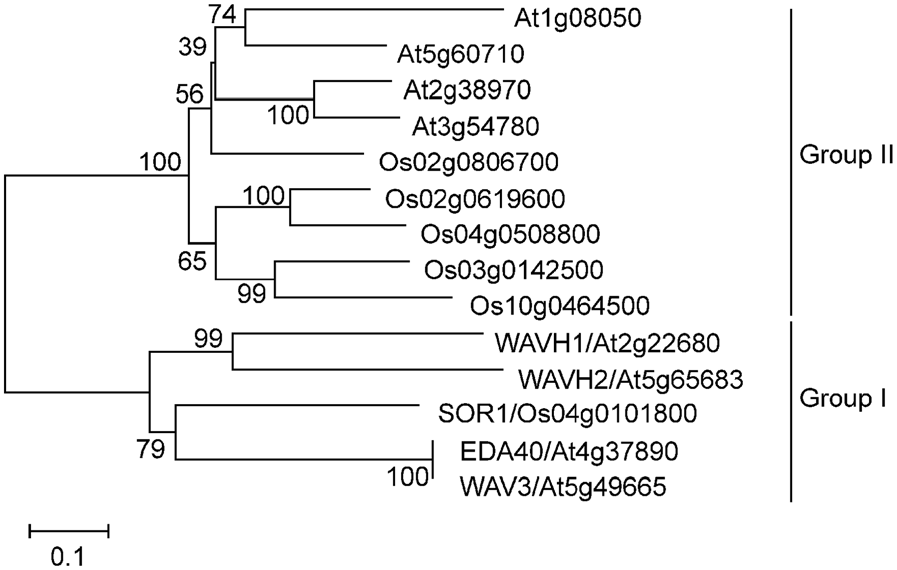
**Phylogenetic relationship of rice and *Arabidopsis *proteins containing both RING and vWFA domains.** Multiple amino acid sequence alignment was carried out and the phylogenetic tree was constructed by using MEGA v. 5.10 (http://www.megasoftware.net) (Tamura et al. 2011). Bootstrap values (1,000 replicates) are shown for each node. Scale bar, number of substitutions per site.

We constructed a phylogenetic tree of rice and *Arabidopsis* proteins homologous to SOR1 (Figure 5). Phylogenetic analysis showed that rice SOR1 and *Arabidopsis* WAV3, EDA40, WAVH1, and WAVH2 form a single monophyletic group (Group I), whereas other proteins form a separate cluster (Group II; Figure 5).

## Discussion

The *sor1* mutant loses the root-specific gravitropic response at an early growth stage. Consequently, the mutant shows soil-surface rooting under a variety of experimental conditions. A 33-bp deletion in the *sor1* mutant gene compromises the gravitropic response; the results of our complementation analysis indicate that the gravitropic response can be restored by a functional *SOR1* gene. The protein encoded by this gene contains the RING and vWFA domains (Figure 4B). Proteins containing the RING domain are involved in auxin signaling (Xie et al. 2002), photomorphogenesis (Hardtke et al. 2002; Holm et al. 2002), plant defense (Serrano and Guzman 2004), brassinosteroid and pathogen responses (Molnȧr et al. 2002), light signaling (Hu et al. 2002), secretory pathways (Matsuda et al. 2001), seed development (Xu and Li 2003), and cellular regulation (Stone et al. 2005). Proteins containing the vWFA domain are involved in transcription, DNA repair, ribosomal and membrane transport, and proteasome regulation (Whittaker and Hynes 2002). The vWFA and RING domains are conserved in yeast, plants, and animals. However, proteins that contain both RING and vWFA domains have been found only in plants, and their physiological functions are unclear. Recently, *wavy growth 3* (*WAV3*), which encodes a protein containing both RING and vWFA domains, was isolated as the causal gene for the *wav3* mutant of *Arabidopsis* (Sakai et al. 2012). The roots of the *wav3* mutant show a short-pitch pattern on inclined agar media and a positive gravitropic response. A total of eight genes encoding proteins with both RING and vWFA domains found in the *Arabidopsis* genome were classified into two subfamilies. Four of these genes, *EMBRYO SAC DEVELOPMENT ARREST 40* (*EDA40*), *WAV3*, *WAV3 HOMOLOG 1* (*WAVH1*), and *WAV3 HOMOLOG 2* (*WAVH2*), belong to the same group. Surprisingly, although the *wav3wavh1wavh2* triple mutant had abnormal root gravitropic response, the single and double mutants were comparatively normal. In the present study, mutation of a single gene in rice, *sor1*, led to the observed deficiency in root gravitropic response (Figures 1, 2, 3). The molecular mechanisms that control root gravitropism are likely to be considerably different between rice and *Arabidopsis*; this difference may be caused by the functional redundancy between the *Arabidopsis* proteins that contain both RING and vWFA domains. In rice, six genes encoding proteins containing both RING and vWFA domains were found (Figure 5). Only one of these proteins, SOR1, belongs to the same group as WAV3, WAVH1, and WAVH2 (Figure 5). Thus, in rice, the control of abnormal gravitropic responses by a single gene (*sor1*) differs from that in *Arabidopsis*, in which three related genes control this response.

Other rice mutants with altered root gravitropic responses have been isolated: RM109 (Hao and Ichii 1999) and *aem1* (Debi et al. 2003, 2005). In the RM109 mutant, root elongation to slantwise direction differs from that in WT. The seminal roots of *aem1* elongate normally, but respond more slowly to gravity than WT. The root gravitropic response likely depends on the difference in auxin concentration between the upper and lower cells due to amyloplast sedimentation in the columella cells of the root tip. Morita and Kyozuka (2007) demonstrated that the auxin transport–related gene *OsPID* is associated with the gravitropic response in rice roots. Ge et al. (2004) identified a regulator of auxin distribution, *OsRAA,* that controls the direction of root growth in rice. The *Arabidopsis* WAV3 family, which has high sequence similarity with rice SOR1, controls the root growth pattern, in particular the gravitropic response, via mechanisms that involve auxin transport and/or signaling (Stone et al. 2005). Further functional studies are needed to determine the relationship between *sor1*, root gravitropism, and hormones.

The *sor1* mutant has relatively thick roots (data not shown) that elongate above and just below the soil surface beginning at the seedling stage. In recent years, root research in cereals has focused on designing root system architecture to optimize nutrient acquisition and enable sustainable production (Hodge et al. 2009; King et al. 2003; Lynch and Brown 2012; Lynch 2013; Rich and Watt 2013). Shallow root systems contribute to efficient absorption and accumulation of nutrients from the soil surface; they are able to reach shallow soil horizons, thus enhancing phosphorus acquisition (Ge et al. 2000; Lynch 2011). Untilled fields gradually accumulate fertilizers and nutrients from decomposing crop residue near the soil surface (Boone 1988). In over-fertilized fields, phosphorus significantly accumulates in the topsoil (Phupaibul et al. 2004). In paddy fields in reclaimed tidal flats, more than 10% of the nutrients supplied by chemical fertilizers are lost due to surface runoff (Cho et al. 2008). Excessive Fe^2+^ content in the reduced layer is produced by waterlogging (Ponnamperuma et al. 1967; Yoshida 1981). The oxidation-reduction potential decreases in paddy soils, because gaseous oxygen is not supplied under swamp condition (Kohno et al. 1995). When oxygen is used up, Fe is reduced; consequently excess Fe^2+^ toxicity is produced in paddy soil solution (Kohno et al. 1995). There is less Fe^2+^ in the upper layers of submerged paddy soils, and soil-surface roots decrease the uptake of iron. Therefore, the mutant *sor1* gene and other genes for soil-surface rooting may be useful for increasing the efficiency of absorption of nutrients accumulated at the soil surface in untilled culture systems and for increasing tolerance to some of the problematic soil types.

Earlier, we identified four QTLs for soil-surface rooting on chromosomes 3, 4, 6, and 7 in three field evaluations (Uga et al. 2012). The major QTL on chromosome 7 was detected in all three field evaluations and by the cup method. The QTL on the terminal region of the short arm chromosome 4 was detected only at the heading stage in 2009. Furthermore, the QTL on chromosome 4 could not be detected by the cup method, so we have been unable to carry out fine-mapping of this QTL. Although further analyses are necessary to determine whether the QTL on chromosome 4 is the same as *SOR1*, this mutant *sor1* gene may be useful for breeding cultivars with enhanced soil-surface rooting.

## Conclusion

The primary roots of the soil-surface rooting mutant lose the root-specific gravitropic response at an early growth stage. *The sor1* (*soil-surface rooting 1*), a rice mutant causing soil-surface rooting and altered root gravitropic response, is allelic to *Os04g0101800* on the terminal region of the short arm of chromosome 4. The 33-bp deletion in *Os04g0101800* is responsible for the mutant phenotypes.

## Methods

### Plant materials

A mutant with primary roots distributed at the soil surface was detected in an M_2_ mutant population regenerated from seed calli of the *japonica* rice (*O. sativa* L.) cultivar ‘Nipponbare’ (Hirochika 1997). This population was grown in 2004 in the paddy field of the Institute of Plant Science and Resources, Okayama University (Kurashiki, Okayama, Japan). To confirm soil-surface rooting in the mutant line, 15 M_3_ progeny from a mutant plant were cultivated in the paddy field of the Experimental Farm Station, Graduate School of Life Sciences, Tohoku University (Kashimadai, Osaki, Miyagi, Japan) in 2005. The seeds of mutant plants with confirmed soil-surface rooting (M_4_) were used for further studies.

For genetic analysis, we used F_2_ populations derived from crosses between the mutant and the *indica* rice cultivar ‘Kasalath’ or between the mutant and WT ‘Nipponbare’. All seeds were soaked in water at 60°C for 10 min and then washed with running tap water for 20 min. Washed seeds were soaked in 0.2% (v/v) plant preservative mixture (PPM; Plant Cell Technology, Inc., Washington, DC, USA) in a 6-cm glass Petri dish. Seeds were incubated at 30°C for 2 d to promote uniform germination.

### Root vertical growth angle

We measured the vertical growth angle of primary roots by using the basket method (Oyanagi et al. 1993; Nakamoto and Oyanagi 1994). Soil containing chemical fertilizer (Nursery culture soil No. 3; Mitsui-Toatsu, Tokyo, Japan: N, 0.7 g · kg^–1^; P, 1.2 g · kg^–1^; K, 0.6 g · kg^–1^) and soil without fertilizer (Shibanome-tsuchi; Kikuchi Industry Co., Ltd., Tochigi, Japan) were mixed in a 1:1 ratio and placed into plastic pots (11 cm diameter × 15 cm height, with a 1-cm diameter hole in the bottom) to a depth of 4 cm. A stainless steel wire basket (6 cm diameter × 3 cm depth; Figure 1G and H) was buried just under the soil surface (open side up) in each pot. The pots were placed inside a plastic container (35 × 60 × 20 cm), and one germinated seed was placed at the center of each basket with the embryo facing upward on the soil in each plant pot. The soil in each pot was watered to maintain a sufficient level of moisture, and the seeds were embedded at a depth of 1 cm. The pots were covered with a vinyl sheet to prevent desiccation until the shoot tip emerged. From the second leaf stage on, the water level in each plastic container was maintained at the soil surface level by using tap water. The plants were grown in a greenhouse at 20–30°C under natural light.

The baskets were dug out when the plants were at the third, fifth, and seventh leaf stages (Figure 1G and H). The soil attached to the baskets was removed carefully without cutting the primary roots. Each basket was divided into four angle areas: < 0°, 0–30°, 30–60°, and 60–90°. The gravitropic and horizontal directions were 90° and 0°, respectively. We counted the number of primary roots emerging from the basket in each angle area.

### Direction of elongation of the primary roots and shoots during seedling development

The direction of elongation was determined by using a bending method. Germinated seeds were sown just beneath the surface of 1.5% agar (Wako Pure Chemical Industries, Ltd., Osaka) in plastic trays (22 × 14 × 4 cm) with the embryos facing upward (Figure 3A). The trays were placed in a growth chamber (Biotron, Nippon Medical & Chemical Instruments Co., Ltd., Osaka) under white light (photosynthetically active radiation equal to 100 μmol/m^2^∙s^-1^) at 25°C and 90% relative humidity. After 18 h, the trays were set on their short sides (Figure 3B). After an additional 10 h, the trays were rotated 90° clockwise (Figure 3C). The direction of seminal root elongation was photographed and measured using Image J software (Wayne Rasband, U.S. National Institutes of Health, Bethesda, MD, USA; http://rsbweb.nih.gov/ij/docs/faqs.html).

### Soil-surface rooting at the seedling stage

For the cup method (Uga et al. 2012), each F_2_ plant was grown in a cylindrical plastic cup (3.7 cm diameter and 4 cm depth; Beaker PP, AS ONE Corporation, Osaka, Japan) (Figure 1C and D). Small holes were made in the bottom of each cup for supplying water. The cups were filled with soil (Rice nursery culture soil No. 3). Forty cups were placed in a stainless steel tray (32 × 25 × 5.3 cm) with drainage holes. Each germinated seed was sown at the center of a cup in a randomized complete block design, and covered with a 1-cm layer of soil without fertilizer (Shibanome-tsuchi). The tray was placed in a large plastic container (44.5 × 32.5× 7 cm). Until the second leaf stage, the water level was maintained at 2 cm above the soil surface from a supply beneath the tray. From the second leaf stage to the time of rooting assessment, the water level was maintained at the level of the soil surface. The plants were grown in a greenhouse at 20–30°C under natural day length. At the third leaf stage, the top layer of soil covering the cups was removed carefully without cutting primary roots that had grown over the edges of cups, and the cups were removed from the tray. The primary roots that had elongated past the edge of the cup were counted as soil-surface roots (Figure 1C and D). The plants were then removed from the cups, and the soil attached to the roots was washed away. The total number of roots was counted for each plant. The soil-surface root ratio for each plant was defined as the number of soil-surface roots divided by the total number of primary roots, and was expressed as a percentage.

For the glass tube method, one germinated seed was sown in a glass tube (1.8 × 18 cm) filled with 10 ml of 0.4% (w/v) agar, and the opening of the tube was covered with aluminum foil. Plants were cultured in a growth chamber as for the direction of elongation assay for 7 d. Plants with roots that elongated on the upper side of the agar surface were considered as the mutant type (Figure 1E), and those with roots that elongated in the agar as WT (Figure 1F).

After evaluating the gravitropic response of F_2_ seedlings from a cross between the *sor1* mutant and WT Nipponbare, we transplanted the seedlings into the center of baskets, so that the coleoptilar node base was embedded into soil at a depth of 1 cm. At the seventh leaf stage, we counted the number of primary roots that emerged from the < 0° angle area (i.e., above the soil surface) and the total number of primary roots, and calculated the soil-surface rooting ratio from these values.

### Mapping of the gene responsible for soil-surface rooting

Seventy DNA markers identified polymorphic between Nipponbare and Kasalath were used for rough mapping (Additional file 1). DNA markers were selected at an average interval of 24.7 cM. The primer sequences and PCR conditions were as described (Temnykh et al. 2000; McCouch et al. 2002).

Seven DNA markers located on the short arm of chromosome 4 were used for fine mapping (Additional file 2). The simple sequence repeat (SSR) markers *RM16254*, *RM551*, and *RM335* were obtained from Gramene (http://www.gramene.org/). The cleaved amplified polymorphic sequence marker *R2373* was obtained from the Rice Genome Research Program (http://rgp.dna.affrc.go.jp/E/index.html). The remaining three DNA markers were designed to detect insertions or deletions by comparison with the genomic sequence databases for Nipponbare (Rice Annotation Project Database; http://rapdb.dna.affrc.go.jp/) and 93–11 (Rice Information System; http://rice.genomics.org.cn).

DNA was extracted from a leaf tip (2 cm) of each F_2_ plant. Each leaf tip was placed in a microcentrifuge tube containing extraction buffer (200 mMTris-HCl [pH 8.0], 250 mM NaCl, 25 mM EDTA, 0.5% [w/v] sodium dodecyl sulfate), and homogenized by using a TissueLyser (Qiagen, Venlo, Netherlands). DNA was precipitated with isopropanol and resuspended in 50 μl of TE buffer (10 mM Tris–HCl [pH 8.0], 1 mM EDTA). The PCR profile involved an initial denaturation of 94°C for 2 min followed by 35 cycles of 94°C for 30 sec, 55°C for 30 sec, 72°C for 30 sec, and a final extension for 4 min at 72°C by using *Taq* polymerase (TaKaRa, Otsu, Japan). The amplified fragments and the products of their digestion by restriction enzymes were analyzed by electrophoresis on 1.5 or 3.0% agarose gels.

### Complementation analysis of the candidate sor1 gene

We used a 6677-bp genomic DNA fragment of Nipponbare that contained the entire *Os04g0101800* coding region, the 2052-bp upstream sequence, and the 1037-bp downstream sequence. The *attB* PCR products were cloned into the pDONR221 vector (Life Technologies, California, USA). The cloned fragment was recombined into pFAST-G01 (Inplanta Innovations, Yokohama, Japan) by using LR Clonase (Invitrogen). The construct was transformed into *Agrobacterium tumefaciens* strain EHA105. Transgenic lines were generated by transformation of the mutant plants with a *Sor1* (wild-type) allele or control empty vector. The seminal root direction of the T_1_ seeds was examined by using the glass tube method. T_1_ seedlings were transplanted into pots, and soil-surface rooting was analyzed by using the basket method.

The absence or presence of the 33-bp deletion in *Os04g0101800* was determined by PCR with specific primers FW (5′-AGCACCTTGGTGGCCTTG-3′) and RV (5′-CCTCGTCATCGCCTCTCTC-3′). PCR was performed by using PrimeSTAR HS DNA polymerase (TaKaRa, Otsu, Japan) in a volume of 10 μl that contained 1 × PrimeSTAR buffer, 200 μM of each dNTP, 0.3 μM of each primer, 0.5 U PrimeSTAR polymerase, and the template. The PCR program was 1 min at 94°C, followed by 30 cycles of 10 s at 98°C and 30 s at 68°C, with a final extension step of 5 min at 72°C. The PCR fragments were analyzed by electrophoresis on 3.0% agarose gels.

### Phylogenetic analysis

Multiple amino acid sequence alignment was carried out and the phylogenetic tree was constructed by using MEGA v. 5.10 (http://www.megasoftware.net) (Tamura et al. 2011). Bootstrap values (1,000 replicates) are shown for each node, and the scale bar indicates the number of substitutions per site. Sequences of putative genes in rice and *Arabidopsis* encoding proteins that contain both vWFA and RING domains were obtained by searching the RAP-DB database (http://rapdb.dna.affrc.go.jp/index.html) and TAIR (http://www.arabidopsis.org/index.jsp), respectively.

## Competing interests

The authors declare that they have no competing interests.

## Authors’ contributions

MM, AM and HH provided the mutant seeds. MM, EH, SN and TS generated F_2_ and F_3_ seeds for genotyping and phenotyping. EH performed the experiments of root assays and molecular analysis. EH, SN, KS, MO, YU, AH and TS designed and discussed the research. EH, KS, MO and TS wrote the manuscript. All authors approved the manuscript.

## Supplementary Material

Additional file 1 **Markers used for rough mapping of the entire genome.** Map distance (Map dist.) is shown as genetic distance (cM) from the 3′ end of the short arm of each chromosome (Chr.). SSR, simple sequence repeat marker; STS, sequence-tagged site marker. Click here for file

Additional file 2 **Markers in the terminal region of the short arm of chromosome 4 used for fine mapping.** SSR, simple sequence repeat marker; CAPS, cleaved amplified polymorphic sequence marker. Click here for file
